# Pathological changes in the lungs and lymphatic organs of 12 COVID-19 autopsy cases

**DOI:** 10.1093/nsr/nwaa247

**Published:** 2020-09-29

**Authors:** Qian Liu, Yu Shi, Jun Cai, Yaqi Duan, Rongshuai Wang, Hongyan Zhang, Qiurong Ruan, Jiansha Li, Lei Zhao, Yifang Ping, Rong Chen, Liang Ren, Xiaochun Fei, Heng Zhang, Rui Tang, Xi Wang, Tao Luo, Xindong Liu, Xuequan Huang, Zhenhua Liu, Qilin Ao, Yong Ren, Jing Xiong, Zhicheng He, Haibo Wu, Wenjuan Fu, Pengnan Zhao, Xinwei Chen, Guoqiang Qu, Yunyun Wang, Xi Wang, Jia Liu, Dongfang Xiang, Sanpeng Xu, Xiaowei Zhou, Qingrui Li, Jinghong Ma, Heng Li, Jie Zhang, Sizhe Huang, Xiaohong Yao, Yiwu Zhou, Chaofu Wang, Dingyu Zhang, Guoping Wang, Liang Liu, Xiu-Wu Bian

**Affiliations:** Department of Forensic Medicine, Tongji Medical College, Huazhong University of Science and Technology, Wuhan 430030, China; Institute of Pathology, Southwest Hospital, Third Military Medical University (Army Medical University), Chongqing 400038, China; Key Laboratory of Tumor Immunopathology, Ministry of Education of China, Chongqing 400038, China; Department of Pathology, Shanghai Jiao Tong University School of Medicine, Shanghai 200025, China; Institute of Pathology, Tongji Hospital, Tongji Medical College, Huazhong University of Science and Technology, Wuhan 430030, China; Department of Pathology, Tongji Medical College, Huazhong University of Science and Technology, Wuhan 430030, China; Hubei Chongxin Judicial Expertise Center, Wuhan 430415, China; Southwest Hospital, Third Military Medical University (Army Medical University), Chongqing 400038, China; Institute of Pathology, Tongji Hospital, Tongji Medical College, Huazhong University of Science and Technology, Wuhan 430030, China; Department of Pathology, Tongji Medical College, Huazhong University of Science and Technology, Wuhan 430030, China; Institute of Pathology, Tongji Hospital, Tongji Medical College, Huazhong University of Science and Technology, Wuhan 430030, China; Department of Pathology, Tongji Medical College, Huazhong University of Science and Technology, Wuhan 430030, China; Department of Pathology, Shanghai Jiao Tong University School of Medicine, Shanghai 200025, China; Institute of Pathology, Southwest Hospital, Third Military Medical University (Army Medical University), Chongqing 400038, China; Key Laboratory of Tumor Immunopathology, Ministry of Education of China, Chongqing 400038, China; Department of Pathology, Wuhan Jinyintan Hospital, Wuhan 430023, China; Department of Forensic Medicine, Tongji Medical College, Huazhong University of Science and Technology, Wuhan 430030, China; Department of Pathology, Ruijin Hospital, Shanghai Jiao Tong University School of Medicine, Shanghai 200025, China; Department of Pathology, Ruijin Hospital, Shanghai Jiao Tong University School of Medicine, Shanghai 200025, China; Institute of Pathology, Southwest Hospital, Third Military Medical University (Army Medical University), Chongqing 400038, China; Key Laboratory of Tumor Immunopathology, Ministry of Education of China, Chongqing 400038, China; Institute of Pathology, Tongji Hospital, Tongji Medical College, Huazhong University of Science and Technology, Wuhan 430030, China; Department of Pathology, Tongji Medical College, Huazhong University of Science and Technology, Wuhan 430030, China; Institute of Pathology, Southwest Hospital, Third Military Medical University (Army Medical University), Chongqing 400038, China; Key Laboratory of Tumor Immunopathology, Ministry of Education of China, Chongqing 400038, China; Institute of Pathology, Southwest Hospital, Third Military Medical University (Army Medical University), Chongqing 400038, China; Key Laboratory of Tumor Immunopathology, Ministry of Education of China, Chongqing 400038, China; Department of Vascular Surgery, Southwest Hospital, Third Military Medical University (Army Medical University), Chongqing 400038, China; Department of Pathology, Ruijin Hospital, Shanghai Jiao Tong University School of Medicine, Shanghai 200025, China; Institute of Pathology, Tongji Hospital, Tongji Medical College, Huazhong University of Science and Technology, Wuhan 430030, China; Department of Pathology, Tongji Medical College, Huazhong University of Science and Technology, Wuhan 430030, China; Department of Pathology, General Hospital of Central Theater Command of PLA, Wuhan 430070, China; Institute of Pathology, Tongji Hospital, Tongji Medical College, Huazhong University of Science and Technology, Wuhan 430030, China; Institute of Pathology, Southwest Hospital, Third Military Medical University (Army Medical University), Chongqing 400038, China; Key Laboratory of Tumor Immunopathology, Ministry of Education of China, Chongqing 400038, China; Department of Pathology, The First Affiliated Hospital of USTC, Division of Life Sciences and Medicine, University of Science and Technology of China, Hefei 230036, China; Institute of Pathology, Southwest Hospital, Third Military Medical University (Army Medical University), Chongqing 400038, China; Key Laboratory of Tumor Immunopathology, Ministry of Education of China, Chongqing 400038, China; Southwest Hospital, Third Military Medical University (Army Medical University), Chongqing 400038, China; Department of Pathology, General Hospital of Central Theater Command of PLA, Wuhan 430070, China; Hubei Chongxin Judicial Expertise Center, Wuhan 430415, China; Department of Forensic Medicine, Tongji Medical College, Huazhong University of Science and Technology, Wuhan 430030, China; State Key Laboratory of Virology, Wuhan Institute of Virology, Center for Biosafety Mega-Science, Chinese Academy of Sciences, Wuhan 430071, China; State Key Laboratory of Virology, Wuhan Institute of Virology, Center for Biosafety Mega-Science, Chinese Academy of Sciences, Wuhan 430071, China; Institute of Pathology, Southwest Hospital, Third Military Medical University (Army Medical University), Chongqing 400038, China; Key Laboratory of Tumor Immunopathology, Ministry of Education of China, Chongqing 400038, China; Institute of Pathology, Tongji Hospital, Tongji Medical College, Huazhong University of Science and Technology, Wuhan 430030, China; Hubei Chongxin Judicial Expertise Center, Wuhan 430415, China; Institute of Pathology, Southwest Hospital, Third Military Medical University (Army Medical University), Chongqing 400038, China; Key Laboratory of Tumor Immunopathology, Ministry of Education of China, Chongqing 400038, China; Hubei Chongxin Judicial Expertise Center, Wuhan 430415, China; Department of Pathology, The First Affiliated Hospital of USTC, Division of Life Sciences and Medicine, University of Science and Technology of China, Hefei 230036, China; Department of Forensic Medicine, Tongji Medical College, Huazhong University of Science and Technology, Wuhan 430030, China; Department of Forensic Medicine, Tongji Medical College, Huazhong University of Science and Technology, Wuhan 430030, China; Institute of Pathology, Southwest Hospital, Third Military Medical University (Army Medical University), Chongqing 400038, China; Key Laboratory of Tumor Immunopathology, Ministry of Education of China, Chongqing 400038, China; Department of Forensic Medicine, Tongji Medical College, Huazhong University of Science and Technology, Wuhan 430030, China; Department of Pathology, Ruijin Hospital, Shanghai Jiao Tong University School of Medicine, Shanghai 200025, China; Research Center for Translational Medicine, Wuhan Jinyintan Hospital, Wuhan 430023, China; Joint Laboratory of Infectious Diseases and Health, Wuhan Institute of Virology and Wuhan Jinyintan Hospital, Chinese Academy of Sciences, Wuhan 430023, China; Institute of Pathology, Tongji Hospital, Tongji Medical College, Huazhong University of Science and Technology, Wuhan 430030, China; Department of Pathology, Tongji Medical College, Huazhong University of Science and Technology, Wuhan 430030, China; Department of Forensic Medicine, Tongji Medical College, Huazhong University of Science and Technology, Wuhan 430030, China; Institute of Pathology, Southwest Hospital, Third Military Medical University (Army Medical University), Chongqing 400038, China; Key Laboratory of Tumor Immunopathology, Ministry of Education of China, Chongqing 400038, China

**Keywords:** COVID-19, SARS-CoV-2, autopsy, histopathology, immune disorder

## Abstract

Systematic autopsy and comprehensive pathological analyses of COVID-19 decedents should provide insights into the disease characteristics and facilitate the development of novel therapeutics. In this study, we report the autopsy findings from the lungs and lymphatic organs of 12 COVID-19 decedents—findings that evaluated histopathological changes, immune cell signature and inflammatory factor expression in the lungs, spleen and lymph nodes. Here we show that the major pulmonary alterations included diffuse alveolar damage, interstitial fibrosis and exudative inflammation featured with extensive serous and fibrin exudates, macrophage infiltration and abundant production of inflammatory factors (IL-6, IP-10, TNFα and IL-1β). The spleen and hilar lymph nodes contained lesions with tissue structure disruption and immune cell dysregulation, including lymphopenia and macrophage accumulation. These findings provide pathological evidence that links injuries of the lungs and lymphatic organs with the fatal systematic respiratory and immune malfunction in critically ill COVID-19 patients.

## INTRODUCTION

Coronaviruses are non-segmented positive-sense RNA viruses that are constantly transmitted from animals to humans [1]. Although most coronaviruses cause mild respiratory diseases in humans, the epidemics of severe acute respiratory syndrome-associated coronavirus (SARS-CoV) and the Middle East respiratory syndrome-associated coronavirus (MERS-CoV) totaled over 10 000 cumulative cases in the past two decades, with mortality rates of 10% for SARS-CoV and 37% for MERS-CoV [2,3]. Since the end of 2019, COVID-19, caused by a novel coronavirus SARS-CoV-2, has caused a global pandemic [2,3]. Although COVID-19 is generally an acute self-limited disease featured with pneumonia, approximately 15% of COVID-19 patients, especially those of an older age and with pre-existing medical conditions, can rapidly develop fatal systematic conditions, such as acute respiratory distress syndrome and severe cardiovascular or renal injuries, among which 5%–6% of cases may progress into critical status with high mortality [4,5].

Thus, the virological, epidemiological and clinical characteristics of COVID-19 have been extensively investigated and the clinical features profiled. However, COVID-19-associated pathological alterations and the underlying translational relevance are still to be elucidated. Recent investigations through full autopsy or minimally invasive autopsy of COVID-19 decedents by us and other laboratories [6–16], revealed that SARS-CoV-2 hijacked angiotensin converting enzyme 2 (ACE2) for entry into target cells [17] to cause severe pathologic changes in the lungs and multiple extrapulmonary organs/tissues. While clinical observations supported that most of the critically ill COVID-19 patients manifested severe immune disorders [4,5], which might result in damages to the lungs and multiple extrapulmonary organs, the changes of immune cell signature in the lungs and lymphatic organs such as the spleen and lymph nodes have not been fully investigated. To profile pathological changes underlying the severe respiratory distress syndrome and immune dysfunction in critically ill COVID-19 patients, we performed systematic autopsy and comprehensive pathological analyses of 12 deceased patients with COVID-19. Our findings provide new evidence on pathological changes associated with COVID-19 and suggest clinical approaches against the disease.

## RESULTS

### Clinical characteristics of the COVID-19 autopsy cases

A total of 12 autopsy cases in this study included seven males (58.3%) and five females (41.7%). The age of the patients was between 51 and 88 years (mean: 70.6 years/median: 72.5 years). All 12 cases had been diagnosed with SARS-CoV-2 infection with pneumonia-like symptoms and abnormalities on chest images when admitted (Table 1). The median course from the onset of symptoms to death was 29.5 days (range: 20–42 days). Clinical symptoms included fever (11/12 [91.7%]), cough (8/12 [66.7%]), sputum production (4/12 [33.3%]) and dyspnea (10/12 [83.3%]). All patients had lymphopenia. Clinical complications included acute respiratory distress syndrome (12/12 [100%]), cardiac injuries or insufficiency (7/12 [58.3%]), acute kidney injury (7/12 [58.3%]), secondary infection (10/12 [83.3%]), sepsis (4/12 [33.3%]), thrombosis (2/12 [16.7%]) and anemia (1/12 [8.3%]). Medical histories of the patients were listed in Table 1.

**Table 1.** Characteristics of the autopsy COVID-19 cases.

Case no.	Age/gender	Survival since symptom onset (days)	Hospitalization (days)	COVID-19 severity (at admission)	Clinical symptoms	Chest imaging	Past medical history	Clinical diagnoses of death causes and complications
1	73/F^a^	35	32	Severe	Fever, cough, dyspnea	Bilateral patchy interstitial infiltrates	Hypertension, COPD^c^	1. COVID-19 (Critical)
								2. MODS^e^ (including ARDS^d^)
								3. Secondary infection
								4. Sepsis
								5. Thrombosis
2	88/M^b^	20	15	Critical	Fever, cough	Bilateral ground-glass changes	COPD,	1. COVID-19 (Critical)
							low back pain	2. ARDS
								3. Secondary infection
3	74/F	30	6	Severe	Fever, cough, dyspnea	Bilateral diffuse patchy interstitial infiltrates	Brain tumor	1. COVID-19 (Critical)
								2. ARDS
								3. Anemia
								4. Secondary infection
4	64/M	30	19	Severe	Fever, cough, sputum production, dyspnea	Bilateral diffuse ground-glass changes	Tuberculosis,	1. COVID-19 (Critical)
							hypertension	2. ARDS
5	56/F	42	16	Severe	Fever, cough, sputum production, dyspnea	Bilateral patchy interstitial infiltrates	Heart disease (unknown),	1. COVID-19 (Critical)
							post-cholecystectomy	2. MODS
								3. Acute kidney injury
								4. Secondary infection
6	76/M	29	4	Critical	Fever, dyspnea	Patchy ground-glass changes in right lung	No notable past medical history	1. COVID-19 (Critical)
								2. MODS (including ARDS)
								3. Sepsis
								4. Thrombosis
								5. Secondary infection
7	72/M	34	20	Severe	Headache, anorexia	Bilateral patchy ground-glass changes	Hypertension, lacunar cerebral infarction	1. COVID-19 (Critical)
								2. ARDS
								3. Secondary infection
8	86/F	23	18	Severe	Fever, cough, sputum production, dyspnea	Bilateral patchy ground-glass changes	Hypertension, coronary heart disease, chronic renal insufficiency	1. COVID-19 (Critical)
								2. MODS (including ARDS)
								3. Secondary infection
9	62/M	31	15	Severe	Fever, cough, dyspnea	Ground-glass changes in lower lobes of both lungs	No notable past medical history	1. COVID-19 (Critical)
								2. ARDS
								3. Acute liver injury
								4. Acute kidney injury
10	51/M	28	6	Critical	Fever, cough, sputum production, dyspnea	White lung	Hypertension, gout	1. COVID-19 (Critical)
								2. ARDS
								3. Sepsis
								4. Cardiac insufficiency
								5. Acute liver and kidney injuries
								6. Secondary infection
11	78/M	27	13	Critical	Fever, dyspnea	Ground-glass changes in lower lobes of both lungs	Hypertension	1. COVID-19 (Critical)
								2. MODS (including ARDS)
								3. Sepsis
								4. Secondary infection
12	67/F	21	14	Severe	Fever, dyspnea	Bilateral ground-glass changes	No notable past medical history	1. COVID-19 (Critical)
								2. MODS (including ARDS)
								3. Secondary infection

^a^F, female; ^b^M, male; ^c^COPD, chronic obstructive pulmonary disease; ^d^ARDS, acute respiratory distress syndrome; ^e^MODS, multiple organ dysfunction syndrome.

### Pathological changes in the lungs

**Figure 1.**
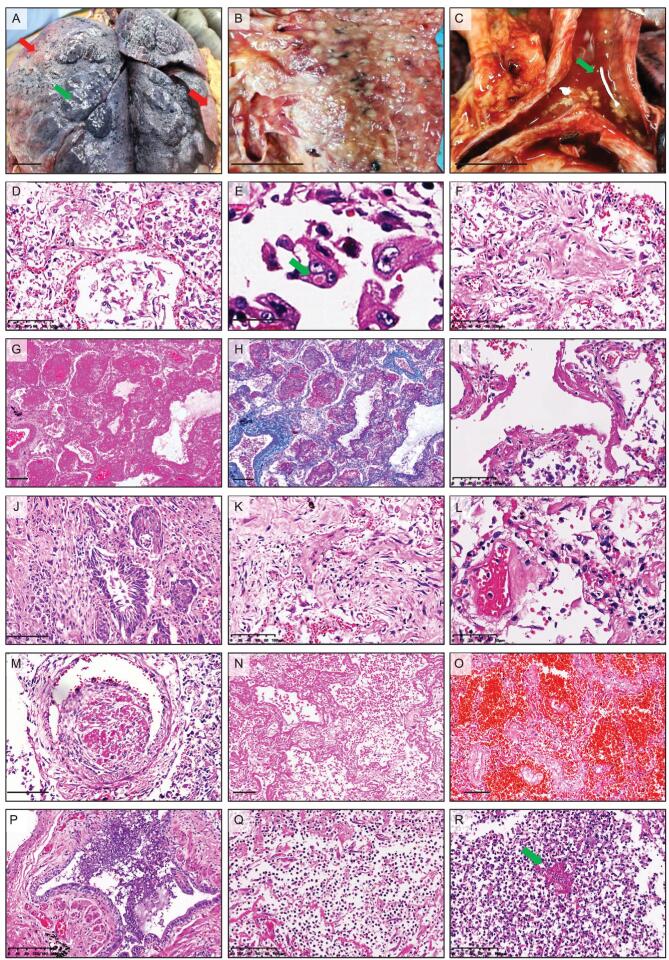
Pulmonary changes in COVID-19 autopsy cases. (A–B) Gross images of the postmortem lungs (Case 10 in A and Case 5 in B) showing the bilateral consolidation (red arrows) and peripheral pulmonary bullae formation (green arrows). (C) Gross images (Case 5) showing the exudate or secretion (green arrows) in the tracheal lumen. (D–E) H&E staining showing the extensive pneumocyte exfoliation and type II pneumocyte hyperplasia (D, Case 4) and the virus inclusion body (green arrows) in the exfoliated type II pneumocytes (E, Case 1). (F–I) H&E staining (F, G, I) and Masson staining (H) showing protein-enriched exudation (F, Case 4), fibric organization (G-H, Case 12) and hyaline membranes (I, Case 5) in alveolar space. (J) H&E staining showing the squamous metaplasia in the bronchioles (Case 7). (K) H&E staining showing the interstitial fibrosis and inflammatory cell infiltration (Case 7). (L) H&E staining showing vasculitis (Case 1). (M) H&E staining showing thrombi in small vessels (Case 9). (N–O) H&E staining showing the coagulation necrosis (N, Case 1) and pulmonary hemorrhage (O, Case 10). (P) H&E staining showing mucous exudate in the bronchioles (Case 3). (Q–R) H&E staining showing the marked inflammatory cell infiltration in the alveoli of COVID-19 patients with secondary bacterial infection (Case 2) or fungal infection (Case 5, fungi are indicated by the green arrow). Scale bar = 2 cm (A–C), 100 μm (D, F, I, J, K, M–O, Q, R), 20 μm (E), 200 μm (G–H, P) or 50 μm (L).

Macroscopic changes of the postmortem lungs included increased weight, focal bilateral consolidation and peripheral pulmonary bullae formation (Fig. 1A and B), which were often overlaid by chronic diseases. Additionally, the trachea and bronchus contained mucosa congestion and increased secretion (Fig. 1C). Ten of the 12 autopsy cases (83.3%) were seen with mild pleural effusion and pleural adhesion. The major pulmonary microscopic features were summarized in Table 2. All 12 autopsy specimens manifested diffuse alveolar damage with moderate to extensive pneumocyte desquamation and hyperplasia (Fig. 1D), but virus inclusion body was rarely identified (Fig. 1E). The alveolar space was filled with protein-rich exudation, fibrosis (Fig. 1F–H) and hyaline membrane formation (10/12 [83.3%]) (Fig. 1I). Squamous metaplasia (7/12 [58.3%]) was found in terminal bronchioles (Fig. 1J). In some areas, there was mild to severe interstitial fibrosis (Fig. 1K) with different extents of inflammatory cell infiltration. Microscopic features of pulmonary vessels included vasculitis (Fig. 1L), mixed thrombi in small vessels (Fig. 1M) and hyaline thrombi in capillaries, alongside hemorrhagic necrosis (Fig. 1N) and focal hemorrhage (Fig. 1O). Dilatation of bronchioles with focal mucous epithelial desquamation and mucous exudation were detected in all 12 autopsy lungs (Fig. 1P). Focal to extensive pulmonary abscess (7/12 [58.3%]) (Fig. 1Q) or fungal pneumonia (3/12 [25.0%]) (Fig. 1R) was observed in patients with bacterial, fungal or multiple infections, and sepsis.

**Table 2.** Pulmonary pathological features in COVID-19 autopsy cases.

	Case 1	Case 2	Case 3	Case 4	Case 5	Case 6	Case 7	Case 8	Case 9	Case 10	Case 11	Case 12
Hyaline membrane	0^a^	1	1	1	1	1	2	1	1	3	2	1
Interstitial fibrosis	3	1	3	3	1	1	3	1	3	2	2	2
Atypical pneumocytes	1	1	1	1	1	1	1	0	1	2	1	1
Thrombi	+	+	+	+	+	+	−	−	+	+	+	+
Pulmonary hemorrhage	2	2	1	1	2	3	2	2	1	2	1	3
Bronchopneumonic changes	3	2	2	2	3	1	2	1	2	2	2	2
Fungal infections	−	−	+	−	+	−	+	−	−	−	−	−
Necrosis	1	1	1	1	3	1	1	1	0	0	1	1

^a^0, not present; 1, mild; 2, moderate; 3, severe; −, negative; +, positive.

### Pulmonary immune signature

**Figure 2.**
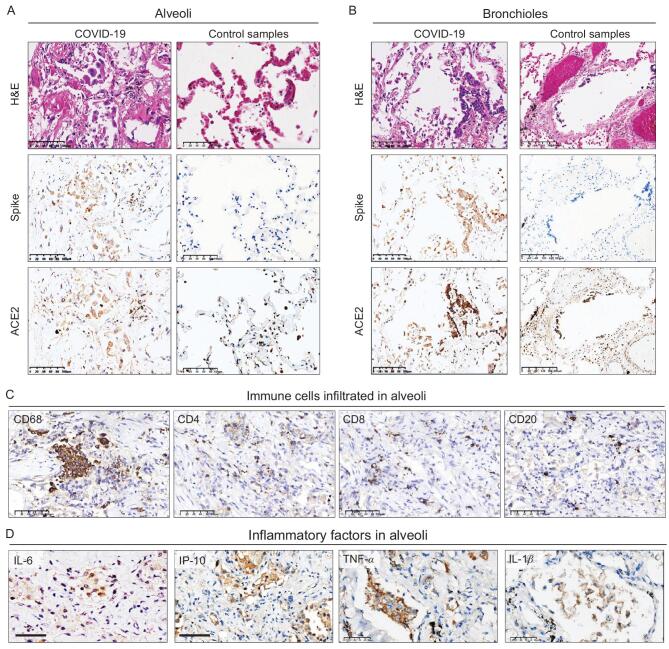
Pulmonary immune signature in COVID-19 autopsy patients. (A and B) IHC staining of SARS-CoV-2 spike protein, ACE2, and the corresponding H&E staining from the same microscopic field using serial sections in alveoli (A, Case 1) and bronchioles (B, Case 2) from COVID-19 cases and control lung tissues from a non-COVID-19 case who died from heart attack (C) IHC staining of macrophages marked by CD68 and lymphocytes marked by CD4, CD8, CD20 in alveoli (Case 3). (D) IHC staining of inflammatory factors (IL-6, IP-10, TNF-α, IL-1β) in serial sections of alveoli (Case 6). Scale bar = 100 μm (A, left panels of B and C), 200 μm (right panels of B) or 50 μm (D).

Immunohistochemical staining showed the SARS-CoV-2 spike protein located in alveolar and bronchial epithelia from the lungs of COVID-19 cases, but not in control pulmonary tissues from a patient who died of heart attack (Fig. 2A and B). Serial sectioning further demonstrated that the SARS-CoV-2 spike protein and ACE2 were co-expressed in alveolar and bronchial epithelia of the COVID-19 cases (Fig. 2A and B), confirming that ACE2-expressing epithelial cells could be the target of SARS-CoV-2 in the lungs. Investigation of pulmonary immune cell signature showed that in contrast with the minor infiltration of CD4- or CD8-positive T lymphocytes or CD20-positive B lymphocytes in alveolar space or the lung interstitial compartment (Fig. 2C), extensive infiltration of CD68-positive macrophages was found in the areas with diffuse alveolar damage in COVID-19 patients (Fig. 2C). Interleukin 6 (IL-6), interferon gamma-induced protein 10 (IP-10), tumor necrosis factor (TNF) α and interleukin 1β (IL-1β) implicated as inflammatory factors associated with COVID-19 progression were detected in the alveoli with diffuse immune cell infiltration (Fig. 2D).

### Pathological changes in the spleen and hilar lymph nodes

**Figure 3.**
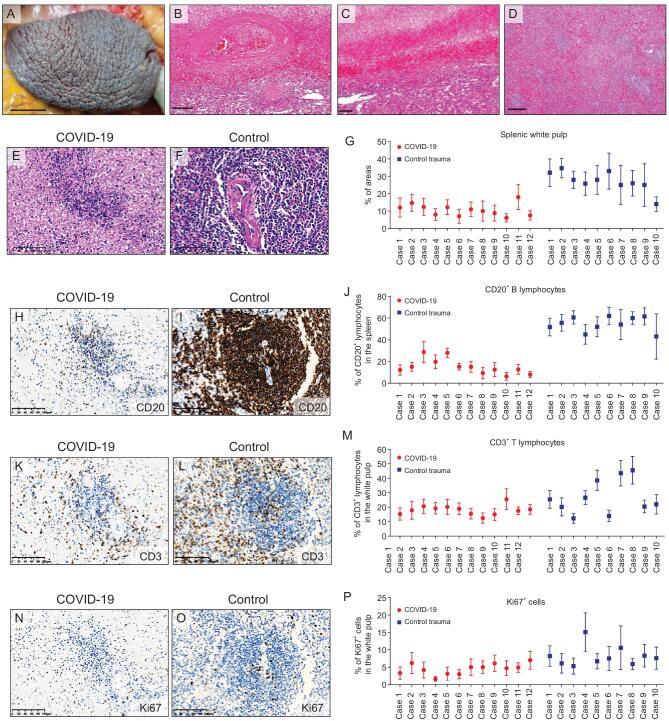
Changes in the spleens of COVID-19 autopsy patients. (A) Gross image showing the contracted spleen with shrinking capsule (Case 6). (B and C) H&E staining showing the mixed thrombi (B) and anemic infarction (C) in the COVID-19 spleens. (D) H&E staining showing the white pulp and red pulp in a COVID-19 spleen (Case 1). (E–G) H&E staining (E and F) and the quantification (G) of the proportion of white pulp areas in the spleens from COVID-19 autopsy cases and control spleens derived from trauma-associated splenectomy. (H–J) IHC staining of CD20 (H and I) and proportion of CD20-positive cells (J) in the spleens from COVID-19 cases and the control trauma spleens. Proportion of CD20-positive cells was calculated as the positive cells versus total cells in at least 10 randomly selected spleen areas under ×200 magnification of microscopic fields. (K–M) IHC staining of CD3 (K and L) and proportion of CD3-positive lymphocytes (L) in the spleens from COVID-19 cases and the control trauma spleens. (N–P) IHC staining of Ki67 (N and O) and proportion of Ki67-positive cells (P) in the spleens from COVID-19 cases and the control trauma spleens. Proportion of CD3- or Ki67-positive cells was calculated as the positive cells versus total cells in at least 10 randomly selected white pulp areas under ×200 magnification of microscopic fields. Data are presented as mean ± SD (G, J, M and P). Scale bar = 1.5 cm (A), 200 μm (B–D) or 100 μm (E, F, H, I, K, L, N and O).

**Figure 4.**
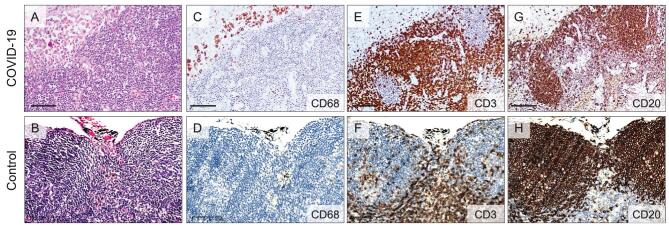
Changes in hilar lymph nodes of COVID-19 patients. (A and B) H&E staining showing the subcapsular sinus expansion in the hilar lymph nodes from COVID-19 autopsy cases (A, Case 9) relative to the control hilar lymph nodes dissected from lung carcinoma patients (B). (C and D) IHC staining showing the increased CD68-positive macrophages in the subcapsular sinus of hilar lymph nodes from COVID-19 autopsy cases (C, Case 9) relative to those in the control hilar lymph nodes (D). (E and F) IHC staining showing the CD3-positive T lymphocytes in the hilar lymph node cortex from COVID-19 autopsy cases (E, Case 9) relative to those in the control hilar lymph node cortex (F). (G and H) IHC staining showing the CD20-positive B lymphocytes in the hilar lymph node cortex from COVID-19 autopsy cases (G, Case 9) and those in the control hilar lymph node cortex (H). Scale bar = 100 μm.

Since a number of severe COVID-19 patients manifested symptoms of immune disorders and lymphopenia, we investigated the pathological changes in lymphatic organs including the spleen and hilar lymph nodes in autopsy cases. The postmortem spleens were generally contracted with shrinking capsules (Fig. 3A). Mixed thrombi, anemic infarction and hemorrhage were found in the contracted spleens (Fig. 3B and C). Moreover, COVID-19 spleens showed atrophic white pulp and relatively enlarged red pulp (Fig. 3D). Lymphoid follicles in white pulp were decreased or absent as compared to control spleens from patients with abdominal trauma necessitating splenectomy (Fig. 3E–G). The proportion of CD20-positive B lymphocytes was reduced in COVID-19 spleens (100%) compared to the control trauma spleens (Fig. 3H–J). Some of the COVID-19 spleens showed decreased CD3-positive T lymphocytes and Ki67-positive cells relative to the control trauma spleens (Fig. 3K–P). Focal lymphocyte apoptosis and macrophage enrichment were

noted in the COVID-19 spleens, which might contribute to the lymphocyte reduction. The predominant changes in the hilar lymph nodes were mildly to moderately expanded subcapsular sinuses infiltrated with CD68-positive macrophages in COVID-19 cases in comparison with the control lung carcinoma cases without tumor metastasis into hilar lymph nodes (Fig. 4A–D). The superficial cortex and paracortex in COVID-19 cases were generally not identifiable with different proportions of necrotic and apoptotic lymphocytes. Interstitial vessels were congested in some COVID-19 cases. The proportions of CD3-positive T lymphocytes and CD20-positive B lymphocytes in the lymph node cortex had no apparent changes (Fig. 4E–H).

## DISCUSSION

COVID-19 patients have been reported to manifest distinct clinical manifestations, disease courses and heterogeneous pathological alterations. Most published papers include limited numbers of COVID-19 autopsy cases. Herein, we systematically evaluated the pathological changes in the lungs and lymphatic organs from 12 COVID-19 autopsy cases. We combined macroscopic and microscopic examinations of the lungs, spleen and lymph nodes, and determined immune cell signature and inflammatory factor production in the lungs. As major targets of SARS-CoV-2, the lungs of COVID-19 decedents showed lesions of diffuse alveolar damage, interstitial fibrosis and exudative inflammation with extensive serous and fibrin exudates, macrophage infiltration and abundant production of inflammatory factors. SARS-CoV-2 and ACE2 were co-localized in the alveoli and bronchioles. The spleen of COVID-19 decedents manifested lymphocyte reduction but macrophage enrichment. Our work together with previous literature provides a comprehensive overview of histopathological changes in COVID-19 patients, implying that acute respiratory distress syndrome, multiple organ injuries,

comorbidities like immune and coagulation disorders, as well as underlying diseases could result in the death of the criticial ill COVID-19 patients [8–11,13,14,16,18,19]. In addition, most of the cases in our cohort were deceased elder patients comparable to published COVID-19 biopsy/autopsy reports [10,16]. Comorbidities in our cohort were also consistent with clinical observational studies [20]. Thus, the corroboration of pathological findings with analyses of the serological and clinical data enables a better evaluation of COVID-19 disease progression and outcome.

A recent study confirmed that SARS-CoV-2 uses coronavirus spike glycoproteins to bind ACE2 for cell entry [21]. Our findings revealed a close association between SARS-CoV-2 infection and ACE2 expression in the lungs, supporting that ACE2-positive cells are targeting cells for SARS-CoV-2 in humans. In agreement with our findings, SARS-CoV-2 infection in ACE2-positive pneumocytes may cause diffuse alveolar damage, hyaline membranes and inflammatory cell infiltration, serving as the pathological basis of blood-air barrier disruption and the generation of lethal hypoxemia. Further investigations are warranted to evaluate the systematic distribution and organotropism of SARS-CoV-2, as well as its association with COVID-19 disease progression and patient outcome.

Lymphopenia has been known as a common manifestation in COVID-19 patients [4]. The decreased proportion of lymphocytes in the spleen shown in our study suggests that SARS-CoV-2 infection may impair the survival of lymphocytes and disrupt lymphocyte-mediated immune reaction. We propose that the following mechanisms may contribute to lymphopenia in COVID-19: (i) the engulfing of lymphocytes by macrophages is frequently observed in the spleen in the postmortem COVID-19 specimens, suggesting that macrophage-mediated phagocytosis may contribute to lymphopenia; (ii) the increase of pro-inflammatory factors, which has been implicated in disrupting the maintenance and proliferation of

lymphocytes, is a common event during COVID-19 progression [22,23]. The use of glucocorticoid in COVID-19 patients may cause immune suppression and lymphocyte reduction. In contract with the reduced proportion of lymphocytes, the proportion of macrophages was significantly increased in alveolar space and the spleen, frequently observed in COVID-19 autopsy cases [18]. These results provide pathological evidence for the development of therapeutics aimed at promoting lymphopoiesis and neutralizing pro-inflammatory signals to prevent cytokine storm in severe COVID-19 patients.

## METHODS

### Autopsy cases and control specimens

The autopsy cases in this study were the first consecutive 12 cases from Jinyintan Hospital, Wuhan, China. All decedents met the diagnostic criteria for COVID-19 and the presence of SARS-CoV-2 in all autopsy cases was confirmed by polymerase chain reaction (PCR) tests. Basic patient information and clinical data were obtained from electronic medical records from Jinyintan Hospital, Wuhan, China as summarized in Table 1. Pulmonary tissues from a patient who died from sudden heart death were used as control for comparison with the lungs from COVID-19 patients. Spleens from patients with blunt or penetrating abdominal trauma necessitating a splenectomy were used as control tissues for comparison with the spleens from COVID-19 patients. The control hilar lymph nodes were derived from lung carcinoma patients receiving hilar lymph node dissection.

### Autopsy procedures

The study was approved by the ethics committee of Jinyintan Hospital, Wuhan, China, and was performed with written consent from patient family members in accordance with the regulations issued by the National Health Commission of China and the Helsinki Declaration. All autopsy procedures were performed in an isolated workspace with adequate negative pressure ventilation in accordance with the Provisional Guidelines on Autopsy Practice for Deaths Associated with COVID-19 in China [19,24]. All staff members had been adequately trained before autopsy examination and were equipped with appropriate personal protective equipment during the practice. None of the staff members involved in this study developed COVID-19. To minimize autolysis, decedents were promptly stored at 4°C after death and the range of the postmortem interval (time of death to time of autopsy) was 4–24 hours. Autopsy materials were collected, fixed in 4% neutral formaldehyde for at least 24 hours and sampled as formalin-fixed, paraffin-embedded (FFPE) tissues for histopathological analyses and SARS-CoV-2 RNA detection.

### Histological, histochemical and immunohistochemical staining

Hematoxylin and eosin (H&E) staining was performed according to the standard procedure, and the results were reviewed by at least two histopathologists independently. Masson staining was performed to evaluate the fibrin and collagen fibers in lung tissues. Immunohistochemical staining (IHC) was performed using the streptavidin-biotin-peroxidase technique with diaminobenzidine. Heat-induced antigen epitope retrieval in ethylenediaminetetraacetic acid (EDTA) buffers (pH: 9.0) or citrate buffer (pH: 6.0) was applied for optimal detection of antigens on FFPE sections. Sections were incubated overnight at 4°C with primary antibodies against CD3 (MXB Biotechnology, Kit-0003, ready for use), CD4 (MXB Biotechnology, RMA-0620, ready for use), CD8 (MXB Biotechnology, MAB-0021, ready for use), CD20 (MXB Biotechnology, Kit-0001, ready for use), CD68 (MXB Biotechnology, Kit-0026, ready for use), IL-6 (Abcam, ab6672, 1 : 250), IP-10 (Abcam, ab9807, 1 : 300), TNF-α (Cell Signaling Technology, 8184, 1 : 20), IL-1β (Cell Signaling Technology, 12 703, 1 : 30), Ki67 (ZSGB-Bio Tech, ZM-0166, ready for use), SARS-CoV-2 spike protein (Sino Biological Inc, 40150-T62-COV2, 1 : 1000) and ACE2 (ZSGB-Bio Tech, TA803841, 1 : 150). The diluent without primary antibodies was used as a negative control. Staining was visualized by Dako REAL™ EnVision™ Detection System (K5007, Dako) followed by counterstaining with hematoxylin. Images were captured using a digital camera (DP73, Olympus) under a light microscope (BX53, Olympus). The histopathological changes in the lungs were defined as positive/negative or semi-quantified for proportions of lesion areas involved in at least 10 randomly selected areas under ×400 magnification of microscopic fields for each specimen by two independent histopathologists. The proportions of CD3, CD20 and Ki67 positive cells in the spleens were calculated as the positive cells versus total cells in at least 10 randomly selected areas under ×200 magnification of microscopic fields.

### Statistical analysis

All statistical analyses were performed using SPSS (Statistical Package for the Social Sciences) version 13.0 software (SPSS Inc.). Categorical variables were described as frequency rates and percentages. Continuous variables were described using the mean and median values. The means for continuous variables were compared using independent group t tests when the data was normally distributed. For unadjusted comparisons, a 2-sided α of less than .05 was considered statistically significant.
